# Development and validation of a questionnaire on 'Satisfaction with dermatological treatment of hand eczema' (DermaSat)

**DOI:** 10.1186/1477-7525-8-127

**Published:** 2010-11-05

**Authors:** Miguel A Ruiz, Felipe Heras, Agusti Alomar, Luis Conde-Salazar, Jesús de la Cuadra, Esther Serra, Francisco Regalado, Ralf Halbach

**Affiliations:** 1 Department of Methodology, School of Psychology, Universidad Autónoma de Madrid, Madrid, Spain; 2 Occupational Dermatology Department, Escuela Nacional de Medicina del Trabajo (National School of Occupational Medicine), Instituto de Salud Carlos III, Madrid, Spain; 3 Dermatology Department, Hospital Sant Pau i Santa Creu de Barcelona. Barcelona, Spain; 4 Dermatology Department, Hospital General Universitario de Valencia, Valencia, Spain; 5 Basilea Pharmaceuticals Iberia S.L., (Fernando el Santo 15), Madrid, (E-28010), Spain

## Abstract

**Objective:**

To develop a self-administered short questionnaire to assess patient satisfaction with medical treatment for hand eczema (dermatitis) with good psychometric properties.

**Method:**

The content of the questionnaire was determined on the basis of clinical consultation with groups of patients, from studying the existing instruments, and from discussions with a panel of seven experts. A first draft version containing 38 items organised in six dimensions was tested on a pilot sample of patients to assess its legibility. The extended version was then tested on a sample of 217 patients of both genders enrolled at 18 hospitals representative of the national distribution. The questionnaire was supplied together with the Morisky-Green compliance questionnaire, the health-related quality of life (HRQL) SF-12 questionnaire, and a visual analogue scale (VAS) of perceived health status to assess concurrent validity. The dimensionality was reduced by means of exploratory factor analysis, and reliability was evaluated on the basis of internal consistency and two halves reliability estimates. Item discriminant capability and questionnaire discriminant validity with respect to known groups of patients (by gender, principal diagnosis, age, disease severity and treatment) were also assessed.

**Results:**

The reduction and validation sample was composed of 54% women and 46% men, of various educational levels with an average age of 43 years (SD = 13.7). Of those who responded, 26% were diagnosed with hyperkeratotic dermatitis of the palms and 27% of the fingertips, and 47% with recurring palmar dyshidrotic eczema. The questionnaire was shortened to a version containing 17 items grouped in six dimensions: effectiveness, convenience, impact on HRQL, medical follow-up, side effects, and general opinion. Cronbach's alpha coefficient reached a value of 0.9. The dimensions showed different degrees of correlation, and the scores had a normal distribution with an average of 58.4 points (SD = 18.01). Treatment satisfaction scores attained correlations between 0.003 and 0.222 with the HRQL measures, and showed higher correlations with the effectiveness (r = 0.41) and tolerability (0.22) measures, but very low correlation with compliance (r = 0.015). Significant differences were observed between some diagnoses and treatments.

**Conclusions:**

The shortened questionnaire proved to have good psychometric properties, providing excellent reliability, satisfactorily reproducing the proposed structure and supplying evidence of validity.

## Introduction

Eczema (dermatitis) affecting the hands has, in many instances, a chronic course and are unresponsive to the various treatments available. If we include mild forms, the annual prevalence may be as high as 10% and affect the patient's life on social, familial, and professional levels to varying degrees [1]. The various therapeutic methods and procedures available for hand eczema trigger variable results; such variability makes comparison across existing studies difficult [2,3]. One of the aspects not taken into account when assessing the effectiveness of these treatments is patient satisfaction with treatment, a parameter that seems to have a substantial influence on quality of life and therapeutic compliance [4-6].

Patient satisfaction is related to all aspects of health care that are important to patient health; including both satisfaction with medical care and with the specific treatment received [7,8]. Patient satisfaction can be conceived of as a pyramid whose base represents satisfaction with medical care; this element includes patient satisfaction with access to medical care, with physician attitude and technical competence, services received, costs, and treatment chosen. The middle tier of the pyramid represents patient satisfaction with overall treatment, including all treatment-related aspects: effectiveness, convenience, undesired effects, follow-up, etc. Finally, at the top of the pyramid, is patient satisfaction with the medication received, representing the evaluation by the patient of the process of taking a medication, including the related outcomes.

Satisfaction with the medication and the medical treatment seems to correlate with patient adherence to treatment [9,10]. It is an indicator of perceived quality that can be used to improve medical care, and it affects patient preferences [11-16]. Moreover, knowledge of the degree of satisfaction with treatment can contribute to predicting therapeutic compliance and help clinicians make better decisions. Therefore, treatment satisfaction is a health indicator that must be considered in both daily clinical practice and biomedical research [17]. Most of the instruments designed to measure patient satisfaction with medical treatment were specific to disease or clinical condition, a situation that not only limited their use, but also did not allow comparison of patient satisfaction with medical treatment across different diseases or medical conditions. Recently, Atkinson and coll. [18] have fine tuned a generic instrument, the Treatment Satisfaction Questionnaire for Medication (TSQM), designed to measure patient satisfaction with drug treatment. TSQM is also available in a short version [19] and another one containing 9 items is currently being drafted. The initial short version includes four dimensions: side effects, effectiveness of the medication, convenience of use, and general patient satisfaction. However, it lacks other dimensions such as patient satisfaction with medical care or the impact of the medication on activities of daily living, both highly important components of patient satisfaction with treatment (particularly with regard to prediction of treatment compliance), since patients consider them attributes of medical treatment [20-23].

Subsequently, other instruments have been developed, such as the SATMED-Q, intended to mitigate the possible limitations of the TSQM and better capture all patient perceptions, evaluating additional dimensions required to effectively measure patient satisfaction with drug treatment. The SATMED-Q is a multidimensional, generic, brief, simple questionnaire that can easily be self-completed by the patient and has good metric properties (reliability and validity) [24]. The questionnaire can be completed by any type of patient, regardless of disease, who are undergoing any prolonged drug treatment.

However, these generic questionnaires seem ill-suited to the situation of patients with eczematous conditions affecting the hands, since the most usual type of treatment uses both emollient creams and oral treatments, a situation that may not be adequately reflected in the existing generic questionnaires. For this reason, we intended to develop a specific measurement instrument able to help guide clinicians in the therapeutic handling of lengthy illnesses toward decisions that, in keeping with patient satisfaction, will favor treatment compliance and effectiveness.

## Method

### Expert panel

The design of the questionnaire began by selecting an expert panel composed of four clinical practitioners specialising in dermatology, a specialist in psychometrics, and a physician specialising in pharmacoeconomics. During the patient enrolment and assessment phase, 15 dermatologists (GEIDAC Group - Grupo Español para la Investigación de la Dermatitis de Contacto y Alergia Cutánea) participated as well. The expert panel was responsible for supervising all study phases, from development to validation of the questionnaire.

The experts performed a bibliographic survey and collected published articles on satisfaction, satisfaction with treatment, satisfaction with services, and health related quality of life (HRQL) in the domains of health sciences and social sciences (Medline, Embase, Current Contents, and Cochrane Library). The existing questionnaires measuring treatment satisfaction were also collected and reviewed, including the SATMED-Q [24] and the TSQM [18], and were used as orientation instruments.

Taking these reviews as a reference point, the expert panel generated an initial pool of questions about the following aspects of drug treatment of skin conditions of the hand: overall satisfaction, effectiveness, convenience of application, undesired effects, cost of treatment, expectations, clinical options available, willingness to recommend the treatment, short-term and long-term consequences, satisfaction with medical care, and impact on daily life.

### Baseline Hypothesis

The expert panel considered the following baseline hypothesis about overall patient treatment satisfaction in relation to their baseline chronic hand eczema (CHE) characteristics.

-	CHE has a similar annual prevalence in both genders, and the hormonal or endocrine system is not involved in its aetiopathogenesis. We therefore do not expect any gender differences.

-	With respect to disease location, even though all eczema studied was located in the hands, eczema affecting the fingertips plays a very strong role in fine sensitivity. Due to the general lack of efficacy of standard treatments, patient treatment dissatisfaction should be higher if fingertips are affected.

-	Finally, the severity of CHE should be the most important factor related to patient treatment satisfaction. Moreover, the different clinical forms (hyperkeratosis, pompholyx, and fingertip CHE) should also influence patient treatment satisfaction. Pompholyx CHE has a worse prognosis than the hyperkeratotic form with respect to treatment efficacy and should therefore influence patient treatment satisfaction.

### Focus groups

Two focus groups were formed (one of eight women and another of eight men, all patients with hand eczema) and underwent a cognitive debriefing in order to elicit patients' ideas about important aspects of their respective treatments, and to find out what treatment-related matters concerned them the most, as well as to obtain additional information on treatment aspects the experts might have overlooked. In all of the groups, the patients were asked about the following topics: (1) The effects of the disease on their daily lives (annoying symptoms) and the limitations imposed by the disease on their daily lives, (2) The importance attributed to the symptoms and limitations, (3) Concerns about the progressive course of the disease or its overall effect on health, (4) Type of medication prescribed, (5) Undesirable side effects of the medication, (6) Benefits of the medication, (7) Compliance with the medication and associated barriers to obtaining full therapeutic compliance.

Both focus groups were enrolled at the Escuela Nacional de Medicina del Trabajo (National School of Occupational Medicine) located in the Autonomous Community of Madrid. All patients were in chronic drug treatment for their condition. Men and women were interviewed separately because it was suspected that hand care habits could differ substantially between the two groups, and discussing them openly might inhibit persons of the opposite sex. The sessions lasted one and a half hours and were moderated by an expert interviewer. The sessions were videotaped and patient comments and responses were transcribed verbatim and summarised, while preserving the participant anonymity.

Both focus groups were homogeneous with regard to patients' CHE and its occupational implications. Also, both included patients in occupations requiring costumer contact (e.g. hairdresser, secretary, waiter, teacher, shopkeeper, nurse), a situation that could entail increased personal perception of CHE-related disability.

Both focus groups reached the conclusion that CHE had a deep effect on their lives, and not only on their jobs, but also on their social and family life. It also had an impact on sleep quality and even on emotional and mental state.

### Generation of items

By combining the contents derived from the initial theoretical framework adopted by the expert panel and the information obtained from the focus groups, dimensions that were considered necessary for inclusion in the questionnaire were defined. An extensive list of items was generated covering, as much as possible, the opinions and perceptions of the patients who had participated in the focus groups. Each of the items was carefully designed to make reference to a single concept, chiefly in a positive sense, avoiding double negatives and ambiguity, and expressed in the first person. A four-point Likert type scale was chosen as the response format, with the following anchor levels: 1 = "Not at all"; 2 = "A little"; 3 = "Enough"; 4 = "A lot".

At least one item was formulated for each of the following aspects: (1) effectiveness of treatment, (2) speed in taking effect, (3) cure expectations, (4) ease/difficulty of administering treatment, (5) convenience of treatment, (6) flexibility of treatment (when and where it can be applied), (7) convenience when not being used (carrying, storing, etc.), (8) patient confidence in his or her ability to use it, (9) length of treatment, (10) satisfaction with treatment planning, (11) impact of treatment on patient's free time, personal relationships, everyday activities, work, and state of mind, (12) information received about treatment, (13) information received about disease, (14) trust in doctor, (15) discomfort with treatment (including side effects and worry), (16) intention of continuing with treatment, (17) general satisfaction with current treatment, (18) recommendation to friends, (19) comparison with another treatments, and (20) treatment costs.

The items initially formulated were evaluated through a discussion and semantic refinement process that produced 41 items grouped in six sections or dimensions:

1. Effectiveness of the medication and its ability to treat the disease and alleviate the symptoms (six items).

2. Convenience of the medication and ease of use (nine items).

3. Impact of the medication on the patient's daily life (seven items).

4. Medical care and follow-up of the disease (five items).

5. Undesirable effects produced by the medication (six items).

6. General opinion, expectations and beliefs about the treatment (eight items).

The final draft of each item was obtained after the expert panel had reached a consensus on it.

### Subjects

The sample of subjects was designed to be representative of the population of patients affected by one of the three most common chronic hand skin conditions: recurring palmar dyshidrotic eczema, dermatitis of the fleshy parts of the hands (with or without palm involvement), and palmar hyperkeratotic dermatitis.

To enroll patients, the researchers offered the GEIDAC group of dermatologists the opportunity to participate. In the end, 19 of those specialists took part in the study (see list of acknowledgements below) and recruited patients at 18 hospitals throughout Spain.

Patients were selected from those requesting an appointment, undergoing epicutaneous tests, and who met the following inclusion criteria: outpatients of both sexes, 18 years of age or older, diagnosed with hand dermatosis using the usual diagnostic criteria used in each researcher's clinical practice, diagnosed at least 12 weeks before inclusion in the study, currently treated for this disease, receiving the same treatment for at least 4 weeks, without allergens or irritants significantly involved in development of the eczema, able to understand the study procedures and answer the health questionnaires associated with this study, voluntarily agreed to participate in the study and signed the informed consent form.

The study has an observational, cross-sectional, multicentre design. With regard to disease treatment, it was conducted under the usual clinical practice conditions. All patients were asked for their informed consent to use their data and to include them in a database. The study protocol was approved by the Sant Pau Hospital Ethics and Clinical Research Committee.

Three different samples were used: (1) *knowledge debriefing sample*: composed of eight men and eight women; (2) *pilot sample*: composed of 13 randomly enrolled patients; and (3) *reduction and validation sample*: defined by applying three representativeness criteria.

The focus groups were sized so as to ensure the active participation of all the members in each of the groups, while still representing a sufficiently broad spectrum of opinions. The size of our pilot sample was considered sufficient to assess the feasibility and pertinence of the questionnaire, as well as to evaluate whether the items were clearly understood by the patients. The size of the reduction and validation sample was determined by following the criterion proposed by Rummel [25], according to which the ratio of subjects to variables should be no less than 4:1. On the other hand, the theoretical framework makes it possible to assume that the treatment satisfaction concept is a multidimensional construct [24,26,27]. Although the number of dimensions used and the degree of relationship among them can vary according to the treatment of interest, the usual practice is to use between five and eight dimensions for assessing all important aspects of the treatment. Moreover, each dimension must contain a minimum of three items in order to be correctly identified [28], although typically a larger number of items (five to ten) is formulated initially, in order to subsequently select those that behave better in the reduction phase.

A reasonable proposal for the first version of the questionnaire may consist of a structure measuring six dimensions, with a minimum of five questions per dimension, which would result in a questionnaire of at least 30 questions. Following the suggestions given above, the advisable sample should contain at least 120 patients. This sample size was considered the minimum recommended size to ensure the metric validity of the study.

Given the number of items in the first version of the questionnaire, and bearing in mind that some subjects could provide non-evaluable responses, it was deemed advisable to select a minimum of 200 patients. This size is usually considered the minimum for obtaining initial scales for correcting a questionnaire in order to ensure its representativeness. Patient selection was random and sequential, and continued until the study quotas indicated above were met.

### Questionnaire reduction

The initial 41-item questionnaire was administered to the pilot sample, together with a brief questionnaire requesting opinions on items not clearly understood, help needed to clarify items, problems found with item wording, time for completion, pertinence of anchor terms, and problems with display format and font size. Problems found were further discussed by the clinician researcher with the patient. The information obtained was used to detect problems with comprehension, pertinence, and legibility of the proposed items. The patients' comments were taken into consideration by the expert panel and integrated when drafting a reviewed version of the questionnaire.

The modified questionnaire, including the contributions made by the pilot sample, was administered to the reduction and validation sample. The information obtained from this sample was used to: (1) verify whether the patients' responses were in line with the structure (dimensions or subscales) proposed by the group of experts, (2) assess the metric properties of the items, (3) reduce the number of questions to a maximum of three per dimension and (4) obtain evidence of the validity of the instrument (see below). The reduction of the questionnaire and the determination of the underlying dimensions were accomplished through a sequence of exploratory factor analyses and by analyzing the internal consistency of the instrument. In the exploratory factor analyses, two extraction methods were used: Principal Components and Maximum Likelihood; and two rotation methods: Varimax (orthogonal) and Oblimin (oblique) [29]. As heuristics for determining the optimum number of factors, Kaiser's K1 rule, the percentage of explained variance, and the size of eigenvalues after rotation [30-32] were applied. Several decision rules were used since it is known that they all tend to underestimate or overestimate the correct number of factors under different conditions [30,33-35]. Internal consistency was assessed by means of Cronbach's alpha reliability coefficient, and taking into consideration the change in the alpha coefficient as items were excluded, one at a time, from the scale [36].

For the process of reducing the length of the questionnaire and analyzing the dimensionality, proposals by Gorusch and Russell were followed [37-39]. Firstly, items suggested as candidates for elimination were those with a clear floor or ceiling effect (items with more than 50% of the responses located in the first or last response category). Secondly, an exploratory factor analysis was conducted with the 41 items of the scale in order to determine the number of underlying factors or dimensions (subscales). Lastly, the dimensionality (factor analysis) and internal consistency (Cronbach's alpha coefficient) of each subscale were analyzed, assuming that each one had to be unidimensional individually.

In this last step, items with lower factor loading in the first dimension or loading in more than one dimension were removed. If a decision needs to be made, those items with the lower contribution to the overall scale alpha were also removed. Items were removed one at a time, until each subscale was left with three items. After each removal, the same analyses were repeated until the unidimensional structure of each subscale proved to be stable and the alpha coefficient did not improve.

Finally, an exploratory factor analysis was performed with all the refined subscales, to ensure that the structure was still stable. All the statistical analyses were performed with the software SPSS for Windows version 16.0.

### Psychometric properties of the final version

The questionnaire was included in a data collection form (DCF) together with relevant clinical information on the patient, socio-demographic information, the Spanish version of the SF-12 quality of life questionnaire [40], a visual analog scale of the patient's current state of health [41,42], the Morisky-Green Compliance Questionnaire [43], the compliance assessment made by the responsible clinician and the tolerability and effectiveness assessments by both the clinician and the patient.

The DCF was administered to the reduction and validation sample. The data collected from this sample were used to: (1) assess the metric properties of the reduced questionnaire and (2) build reference scales for the Spanish population.

The following metric properties were studied for the final questionnaire: (1) *feasibility*: completion time, floor and ceiling effects, and percentage of missing responses for each item; (2) *reliability*: internal consistency was assessed using Cronbach's alpha coefficient, and the Pearson correlation coefficient between items and between each item and the total score; reliability was also estimated by applying the "two halves" method (stability), correlating the scores of the subscale formed by the even-numbered items with the subscale containing the odd-numbered items, and by means of the intraclass correlation coefficient [44-46]; (3) *content validity*: this property was ensured by the active participation of the expert group in the entire content selection and question formulation processes, and by consultation with the patients in the two focus groups; additionally, agreement among six referees was assessed regarding the assignment of items to dimensions as measured by the Rovinelli and Hambleton coefficient [47]; (4) *structural validity*: the structure in dimensions of the responses obtained with the final questionnaire was established through exploratory and confirmatory factor analysis; in both types of analysis, the aim was to test the dimensional structure of the final scale and the location of each item in its respective theoretical dimension; (5) *concurrent validity*: the scores of the reduced version of the questionnaire were correlated with the summary scores of the SF-12 quality of life questionnaire, with the scores of the Morisky-Green Compliance Questionnaire and with the state of health assessment; (6) *discriminant validity*: the ability of each item to discriminate between the 25% of the subjects with the lowest scores and the 25% with the highest scores (created based on the scores on the overall scale) was analyzed, as well as the ability of each scale and of the overall scale to discriminate between groups of patients formed based on the effectiveness and tolerability assessments conducted by the clinicians and by the patients. All the analyses were performed with the SPSS for Windows version 16.0 and AMOS 7.0 software applications.

## Results

### Focus groups

The two focus groups provided coinciding results. Although the patients know that hand dermatoses require ongoing treatment, such treatment is often abandoned as soon as the symptoms disappear. The symptoms are bothersome and highly incapacitating, affecting HRQL (work, social relations, family life, and psychological state). The perception is that the treatments are not very effective, and self-medication is frequent. Patients see the oral forms as more powerful and convenient than creams and gloves, although taking the oral medications often requires the use of gastro protectants. A suitable treatment improves the patient's self-image, self-confidence, quality of life, and mood. They indicate that the medical information is scant and not very specific. There is a fear of transmitting the disease to other people. The majority of patients feel that support groups can benefit them. For women, the appearance of their hands has a greater impact on their self-image and intimate relationships.

The information gathered from these groups enabled us to confirm that no contents that were important to the patients had been obviated. It also made us aware of aspects of the treatment that clinicians do not notice.

### Pilot questionnaire

The sample used during the pilot phase was composed of 13 patients, 46% of whom were women, with an average age of 43 years (SD = 14.6) and an age range from 23 to 61 years. The average time required to complete the questionnaire was 8 min. 45 sec. (SD = 4 min.); the fastest respondent completed it in 3 min. and the slowest in 16 min.

One question (number 38) had to be discarded due to a formatting error in the original. All questions showed variability in the responses and proved to be sensitive to differences of opinion among patients. Except for one subject who left one question blank and three others who left two different questions blank, all respondents answered all of the questions. The questions with omitted answers were numbers 5, 34, 40, and 41. None of the patients needed help in responding and five (39%) encountered some difficulty in understanding the questionnaire. The comments showed that some questions were considered "silly" (numbers 13 and 40) and that patients lack the professional know-how to evaluate the existence of other treatments and are not qualified to recommend treatments. In view of the comments, the following questions were eliminated: question 13 "I am happy with the total length of the treatment (for example: one week, one month, etc.)", question 40 "I believe that there are better medications than the one I am taking", and question 41 "I would advise a person with my symptoms to go to the doctor to try the same treatment".

### Questionnaire reduction

The reduction and validation sample was finally set at 213 analyzable patients, whose average age was 43 years (SD = 13.6) and whose ages ranged from 19 to 83 years. Of this sample, 59% were women. The majority was Caucasian (99%). The distribution by educational level was homogeneous, except for the stratum with no formal education, which comprised only 1% (see Table 1). With regard to the pathologies studied, 47% presented recurring palmar dyshidrotic eczema, 24% had dermatitis of the fleshy parts of the hands (with or without palm involvenement), and 29% had palmar hyperkeratotic dermatitis, with an average disease duration of 54 months (SD = 73.1).

**Table 1:** Demographic characteristics of patients included in study

Variable	Item reduction sample (n = 213)
Age: mean (SD)	43.42 (13.57)
Sex, female: n (%)	124 (58.5%)

Race	
Caucasian	210 (98.6%)
African	2 ( 0.9%)
Other	1 ( 0.5%)

Education	
Illiterate	1 ( 0.5%)
No high school diploma	53 (24.9%)
High school graduate	50 (23.5%)
Professional training diploma	53 (24.9%)
College graduate	55 (25.8%)
Unknown	1 ( 0.5%)

Number of patients by disease	
Palmar hyperkeratotic dermatitis	62 (29.1%)
Dermatitis of the fleshy parts of the hands	52 (24.4%)
Recurring palmar dyshidrotic eczema	99 (46.5%)
	
Disease history (months)	53.82 (73.15)

Table 2 shows the internal consistency results before (initial scale, 38 items) and after (final scale, 17 items) item reduction. The values of the Cronbach alpha coefficient (above 0.82 for all final version subscales) indicate good internal consistency, except for the Convenience subscale, which only attained a value of 0.7. Table 3 reports the descriptive statistics for each subscale.

**Table 2:** Internal consistency of subscales

	Number of items		Cronbach's alpha		% variance explained*
		
Domains	Initial	Final	Initial	Final	
Treatment effectiveness (TE)	6	3	0.884	0.828	75%
Convenience of use (CU)	6	2	0.661	0.698	77%
Impact on activities of daily living (ID)	7	3	0.817	0.881	81%
Medical care (MC)	5	3	0.947	0.922	87%
Undesirable side-effects (UE)	6	3	0.872	0.929	88%
General satisfaction (GS)	8	3	0.765	0.882	81%

Overall score	38	17	0.923	0.904	83%

* Percentage of variance explained by the first factor in each subscale.

**Table 3:** Descriptive statistics for the DermaSat scales and overall score

	N						% cases	
							
	Valid	Missing	Mean	SD	Min	Max	Lower cat	Upper cat
Treatment effectiveness (TE)	213	0	49.69	26.38	0	100	4.7	5.2
Convenience of use (CU)	213	0	63.85	21.35	0	100	1.4	12.2
Impact on activities of daily living (ID)	211	2	49.16	26.85	0	100	8.9	6.1
Medical care (MC)	213	0	64.50	27.51	0	100	2.8	23.9
Undesirable side-effects (UE)	212	1	74.32	27.72	0	100	3.8	39.4
General satisfaction (GS)	211	2	48.92	26.10	0	100	8.5	3.8
Overall score	213	0	58.10	18.16	12.5	100	0.5	1.4

SD = Standard Deviation, Min = Minimum, Max = Maximum, Lower cat =% of cases in lower response category, Upper cat = % of cases in upper response category.

Factor analysis performed with the 38 items indicated that admissible solutions varied between four and nine common dimensions underlying the correlation matrix. The individualised analysis of the theoretical dimensions supported the preliminary unidimensional hypothesis except in the cases of convenience and general opinions. The clustering of aspects relative to convenience gave rise to a dimension corresponding to the aspects most directly related to convenience of use, and another dimension involving the aspects relative to overload or negative aspects of compliance. Since the negative aspects of treatment overload showed a floor effect, only the positive aspects were kept in this dimension. Because the clustering of general opinions segregated a dimension that comprised aspects regarding the financial expense of the rest of the general satisfaction items, only the general satisfaction items were kept. Once the questionnaire had been reduced, the percentage of variance explained by the first dimension of each reduced subscale supported the unidimensional character of the subscales (see Table 2).

### Scaling and dimensionality

The results of the factor analysis of the reduced scale (see Table 4) suggested the presence of four to six dimensions. The goodness-of-fit test for the maximum likelihood solution (chi-squared = 51.8; gl = 49; p = 0.364) supports the six-dimension solution, adequately explaining correlations among the items. Whereas the fifth and sixth initial eigenvalues presented values below one, after orthogonal rotation all the dimensions attained eigenvalues above one. The six-dimension solution is meaningful and accounts for 83.2% of the available variance. The commonalities of the variables ranged from 0.749 to 0.889.

The exploratory factor solution output (17 items, six dimensions, oblimin rotation), shows that all items preferably load in their corresponding theoretical dimension (see Table 4). Only some items of the *treatment effectiveness *and *general satisfaction *dimensions show loadings below 0.80. In fact, a considerable correlation is observed among the *treatment effectiveness*, *general satisfaction*, and *impact on daily activities *dimensions. The existence of correlations among the dimensions justifies the possibility of creating a single overall summary score. (The Spanish final version of the questionnaire can be found in Additional file 1 and an English version in Additional file 2).

**Table 4:** Validation sample: Exploratory factor analysis solution (oblimin rotation)

	Factors					
	
DermaSat	GS	UE	MC	CU	ID	TE
**Treatment effectiveness (TE)**						
- Relief of symptoms	-.054	-.021	-.036	-.032	-.032	**.968**
- Feel better	.166	.017	.045	.073	.237	**.602**
- Disease under control	.313	-.006	-.129	.105	.079	**.504**
**Convenience of use (CU)**						
- Ease of medication use	.047	-.055	-.010	**.860**	.012	-.097
- Convenient schedule	-.077	.021	-.030	**.877**	-.016	.079
**Impact on activities/daily living (ID)**						
- Leisure activities	.040	.085	.046	.104	**.876**	.011
- Everyday activities	-.105	-.106	-.073	-.094	**.897**	.068
- Better mood	.133	-.017	-.059	.019	**.795**	-.035
**Medical care (MC)**						
- Disease information	-.015	.033	**-.923**	.031	.018	.022
- Treatment information	.032	-.014	**-.917**	-.040	.084	-.052
- Treatment effects information	.020	-.011	**-.912**	.048	-.070	.044
**Undesirable side-effects (UE)**						
- Impact on job activities	.048	**.940**	.067	.044	-.040	.015
- Impact on leisure activities	-.069	**.938**	-.062	-.020	.072	-.015
- Impact on activities of daily living	.019	**.923**	-.013	-.055	-.039	-.001
**General satisfaction (GS)**						
- At ease with treatment	**.630**	-.056	.073	.115	.203	.197
- Better existing choice	**.927**	.008	-.118	-.040	-.039	-.050
- Satisfied with treatment	**.518**	-.018	-.083	.026	.223	.281

Eigenvalues	7.17	2.65	1.75	1.27	0.69	0.61
Percentage of variance explained (%)	42.20	15.60	10.31	7.44	4.05	3.61

	**Factor correlation matrix**					

Undesirable side-effects (UE)	-.049					
Medical care (MC)	-.460	.044				
Convenience of use (CU)	.272	-.193	-.233			
Impact on activities/daily living (ID)	.547	-.214	-.359	.259		
Treatment effectiveness (TE)	.483	-.121	-.288	.233	.605	

Note: item labels have been shortened to fit the table.

### Psychometric properties of the final version

#### Feasibility

The blank response rate in the validation sample (213 patients) was quite small: 92.5% of the patients completed all the questions in the reduced questionnaire. Of the patients who omitted one or more answers, 11 left one question blank, three left three questions blank, and two left six questions blank. As a comparison, the complete response rate to the SF-12 was 92.0%, with one to eight responses left blank.

For all the items, the responses were distributed over all the proposed response categories (from "not at all" to "a lot"). Except for the *undesirable side-effects *subscale, the response distribution is either centered on the central categories or shows a slight negative asymmetry, and all the distributions are unimodal. The *undesirable side-effects *subscale accumulates the responses in the lower part of the scale: between 45.8% and 48.6% of the responses fall into the "Not at all" category, but in no case does the percentage exceed 50%, which indicates a slight floor effect. Among the discarded questions, the maximum floor effect was presented by the question referring to having to see the doctor due to the unwanted effects, with an accumulation of 62% of the answers in the "Not at all" category. The question with the greatest ceiling effect was the one referring to how the doctor encourages the patient to continue with the treatment, which accumulated 36% of responses in the "A lot" category.

#### Reliability

The internal consistency estimation (Cronbach's alpha coefficient) with the validation sample surpasses the value 0.81 for all subscales (see Table 2), although it was slightly lower for the *Convenience *subscale. A value of 0.923 was obtained with the total scale. The first eigenvalue is markedly higher than the second one in all subscales, and the first dimension of each subscale accounts for a percentage of variance ranging from 75% to 88%, all of which indicates that the subscales behave as unidimensional.

The stability of the scale was assessed by correlating two item subsets, each composed of half of the items. The correlation between the two forms (two halves) reached a value of 0.892, the estimation of the reliability of the questionnaire based on the Spearman-Brown split-half approach was 0.943, and the intraclass correlation coefficient (ICC) was 0.904, with a 95% confidence interval of [0.884-0.923].

By adding up the direct scores of the items, a total score is obtained ranging from 17 to 68. In order to transform the total score into a metric with a minimum at 0 and a maximum at 100 (the most intuitive and easy to interpret metric), the following formula can be used:

$$Y^{\prime }=\frac{Y_{obs}-Y_{\min}}{Y_{\max}-Y_{\min}}\times 100=(Y_{obs}-17)\times 1,961$$

Where *Y*_max _= 68 (maximum total score); *Y*_min _= 17 (minimum total score); *Y*_obs _= total score obtained by the patient, *Y' *= transformed score. A similar formula can be used to change the metric of each dimension.

The total scores in the new metric are distributed symmetrically and normally (Kolmogorov-Smirnov = 0.822; p = 0.508) with a mean of 58.10 and a standard deviation of 18.16. The median is 58.82. The minimum score observed was 12.50 and the maximum was 100.

To analyze the discriminant capability of each item considered individually, two groups of patients were created on the basis of the scores obtained on the total scale. The first group was formed with the 25% of the patients with the lowest scores; the second contained the 25% of the patients with the highest scores. The comparisons between these two groups by means of Student's *t*-test produced significant differences in all items (*t*(99) > 5.13, and *p *< 0.0005 in all cases).

#### Content validity

Content validity was ensured by studying the existing bibliography on the subject, consulting the patients who participated in the focus groups, and ratification by consensus of the expert panel.

#### Structural validity

**Figure 1:**
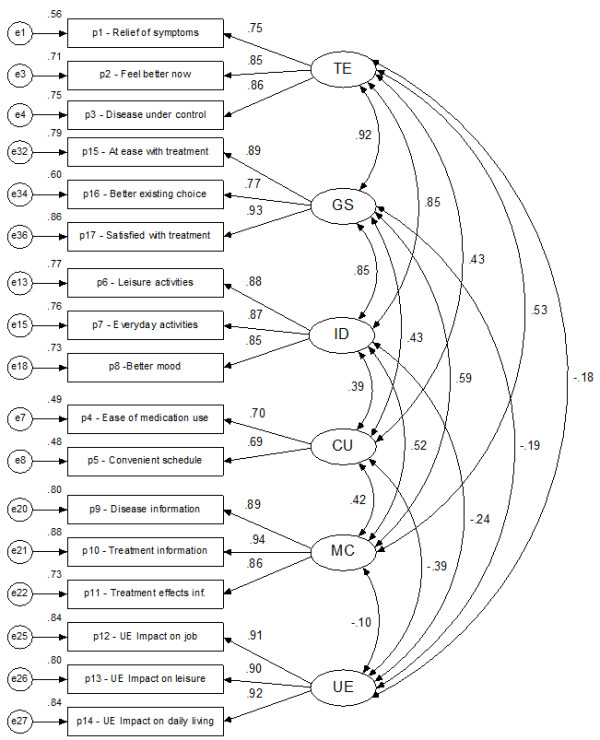
**Confirmatory factor analysis, standardized estimates**. TE = Treatment Effectiveness, GS = General Satisfaction, ID = Impact on Activities of Daily Living, CU = Convenience of Use, MC = Medical Care, UE = Undesired Side-Effects.

Although the factor structure resulting from the scaling analysis provides evidence on how the items cluster together in the concepts they are intended to measure, a confirmatory factor analysis was conducted, imposing the theoretical structure and re-estimating item loadings. Figure 1 shows the results of the confirmatory estimation of the theoretical structure proposed for the questionnaire, using the generalised least squares method. All loadings were significant (*p *< 0.001) as were all the correlations between factors (*p *< 0.05) except for the correlation between *undesirable side-effects *and *satisfaction with medical care *(p = 0.217). The goodness-of-fit statistics indicate a good or very good fit: GFI = 0.918; AGFI = 0.880; CFI = 0.887; RMR = 0.047, χ^2^/gl = 1.312 and RMSEA = 0.040. Even the chi-square goodness-of-fit test makes it possible not to reject the null hypothesis (with α = 0.01) stating that the correlation matrix can be correctly reproduced with the theoretical structure (χ^2 ^= 136.4; gl = 104; p = 0.018), a desirable but infrequent output with sample sizes like the one used. However, the estimated correlations among the three dimensions were extremely high, especially the correlation between *effectiveness *and *general satisfaction *(*r *= 0.92), *impact on daily living *and *general satisfaction *(*r *= 0.85), and *effectiveness *and *impact on daily living *(*r *= 0.85). By contrast, the dimension showing the greatest independence was *undesirable side-effects*, which presented low correlations with respect to the dimensions of *medical care *(*r *= -0,10), *effectiveness *(r = -0,19), and *general satisfaction *(*r *= -0,18).

#### Concurrent validity

The average scores in the components of the SF-12 were significantly lower (p < 0.001) than the theoretical mean of each dimension (50 points); furthermore, the mean in the physical component was slightly lower (mean = 44.9; SD = 9.0) than in the mental component of the SF12 (mean = 46.01; SD = 11.9), although the difference between the two components was not significant (p = 0.292). The mean on the VAS of perceived sate of health was 69.7 points (SD = 20.4), achieving scores between a minimum of 5 and a maximum of 100.

The correlations of the DermaSat dimensions with the quality of life measurements were low. Only the *effectiveness *dimensions correlate with the physical component (r = 0.148; p = 0.038), along with the dimension of (absence of) *undesirable side-effects *with respect to the physical component (r = 0.167; p = 0.019) and the mental component (r = 0.231; p = 0.001). The overall score correlated only with the physical component (r = 0.151; p = 0.035). Correlations with the perceived state of health VAS were higher: with the dimensions of *effectiveness *(r = 0.238; p = 0.001), *impact on daily living activities *(r = 0.148; p = 0.034), *undesirable side-effects *(r = 0.140; p = 0.044), *general satisfaction *(r = 0.186; p = 0.008) and with the overall score (r = 0.221; p = 0.001). No significant correlations were observed of the DermaSat scores with the Morisky-Green compliance score (See Table 5).

**Table 5:** Correlations of DermaSat dimensions with other PRO (SF-12, VAS, Compliance)

	SF-12			
			
DERMASAT	Physical Component	Mental Component	Perceived Health VAS	Morisky-Green
Treatment effectiveness	.148*	-.012	.238**	.059
Convenience of use	.080	.042	.114	-.113
Impact of daily activities	.138	-.019	.148*	.080
Medical care	-.030	-.123	.073	.030
Undesirable side-effects	.167*	.231**	.140*	-.071
General satisfaction	.119	-.004	.186**	.038
Total score	.151*	.028	.221**	.018

* *p *< 0.05, ** *p *< 0.01.

The correlations of the SF-12 dimensions with the VAS scale were slightly higher (physical component: r = 0.429; mental component: r = 0.319) and were significant in both cases (p < 00.1). The compliance score correlated significantly with the physical component (r = 0.177; p = 0.014) and with the VAS scale (r = 0.177; p = 0.011).

#### Construct validity

An important aspect enabling the interpretation of the scores of a scale is the assessment of its relationship with other patient measurements which, from a theoretical standpoint, can be expected to be related to the concept we wish to measure. In this regard it was found that only the scores of the DermaSat effectiveness dimension correlate with the clinician's assessment of patient compliance (r = 0.213; p = 0.002). The highest correlations of the DermaSat dimensions were observed with patient's assessments of treatment effectiveness followed by the size of correlations with clinician's assessments of effectiveness; a significant correlation was observed between clinician assessment and scores in *effectiveness *(r = 0.482), *impact on daily activities *(r = 0.332), *general satisfaction *(r = 0.303), and overall score (r = 0.345) (p < 0.001 in all these cases). The correlation between clinician and patient assessments with regard to effectiveness was very high (r = 0.672; p < 0.001) and the pattern of correlations of DermaSat dimensions with patients assessments was similar to the pattern observed for clinician assessments (Table 6).

**Table 6:** Correlations of DermaSat dimensions with other patient outcomes

	Compliance		Effectiveness		Tolerability	
	
DERMASAT	Patient	Clinician	Patient	Clinician	Patient	Clinician
Treatment effectiveness	.059	.213**	.571**	.482**	.238**	.299**
Convenience of use	-.113	-.028	.121	.050	.144*	.073
Impact on daily activities	.080	.091	.426**	.332**	.128	.210**
Medical care	.030	.076	.105	.107	.023	.071
Undesirable side-effects	-.071	-.070	.055	.094	.053	.126
General satisfaction	.038	.088	.400**	.303**	.088	.129
Total score	.018	.095	.413**	.345**	.156*	.225**

* *p *< 0.05, ** *p *< 0.01.

The clinician assessments of treatment tolerability correlated significantly with the scores of the dimensions of *effectiveness *(r = 0.299) and *impact on daily activities *(r = 0.210), and with the overall score (r = 0.225) (p < 0.002 in all cases), but did not attain significance with the dimensions of *undesired side-effects *(r = 0.126; p = 0.066). The correlations between the clinician and patient assessments were high (r = 0.570; p < 0.001), but the correlations of patient assessments with DermaSat dimensions were somewhat lower.

#### Validity with respect to known groups

Another aspect of the construct validity is the ability of the scale to discriminate between groups of patients with different characteristics. When we compared the scores in the DermaSat dimensions between known groups, we observed significant differences between sex groups only in the convenience dimension (d = 8.05; p < 0.009) with higher satisfaction with the treatment convenience in the women's group (mean = 67.3; SE = 1.6) than in the men's group (mean = 59.3; SE = 2.6).

**Figure 2:**
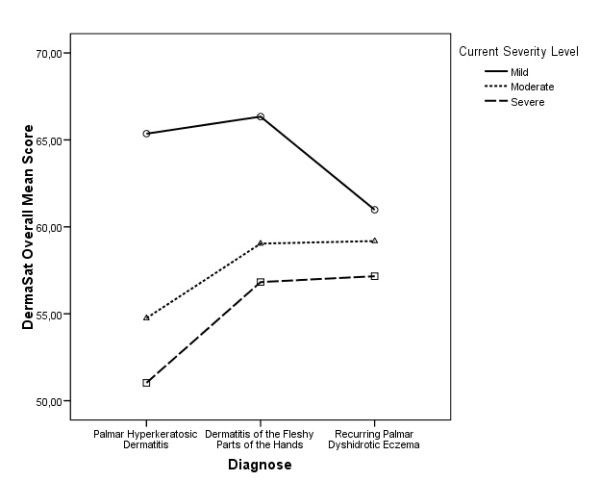
**DermaSat overall average values by disease and severity level**.

When comparing the overall scores by type and severity of pathology (Figure 2), we did not observe significant interaction between the two classification criteria (p = 0.837), nor did we find significant differences by diagnosis type (p = 0.621), but the differences by level of severity were found to approach significance (p = 0.063), so the severely affected group of patients show significant differences with respect to the slightly affected group (p = 0.05).

#### Scales

Table 7 offers the scaling correction values. These values make it possible to translate the scores observed in the 0-100 metric to the corresponding decile in the normative sample. For example, a patient with a total score of 78 points will fall into the 9^th ^decile group, which means that 90% of the patients in the population show the same or less overall satisfaction than the patient.

**Table 7:** DermaSat total deciles

	Total	
	
Deciles	Min.	Max.
1	0.00	0.00
2	11.11	22.22
3	33.33	33.33
4	33.33	33.33
5	44.44	44.44
6	55.56	55.56
7	66.67	66.67
8	66.67	66.67
9	77.78	77.78
10	88.89	100.00

## Discussion

The aim of this study was to develop a new specific instrument that would be able to measure satisfaction with treatments for hand eczemas, especially CHE, to test its psychometric properties, and to provide data regarding its validity.

The findings of this study show that the DermaSat Questionnaire is a valid, reliable, and feasible instrument for use in routine medical practice, both as a unidimensional instrument for comparing patients (using the total score), and when the clinician wishes to explore patient satisfaction with the various facets of treatment (for which the subscales of the instrument have also proven valid and reliable).

CHE is a disease that is difficult to treat, which can severely impact quality of life. As demonstrated in the literature, the impact can be as severe as in asthma or psoriasis [48]. Currently, few effective therapeutic options are available to help patients. Furthermore, the impact of CHE on patients' lives is underestimated. Clearly, CHE is not life-threatening but, since it is centered on the hands, it may affect relationships and social integration, and even cause rejection.

Several treatment satisfaction questionnaires have been developed to date and may be used in patients with CHE. Due to the very specific circumstances of CHE, none of the existing instruments was deemed specific enough to give clinicians a reliable tool for evaluating treatment satisfaction. It was felt that there was a clear need to develop a specific CHE treatment satisfaction questionnaire. The specificity of this questionnaire will allow the clinician to better understand the patient's personal experience with current treatments, based on an objective tool. Depending on the outcomes, the clinician will be able to change and adapt the treatment algorithm.

The results obtained reveal that the DermaSat has very good metric properties. From the standpoint of *feasibility*, the response rate is highly satisfactory (nearly all patients answered all questions) and the time required to administer it was brief (under eight minutes on average), which makes it highly feasible for use at any level of health care, particularly at the primary level, where the time available for care is usually short.

With regard to the *reliability *of the questionnaire, both internal consistency and two halves reliability methods attain higher values than the accepted standard minima [49] with respect to the total score and also to the individual subscales. And the analysis of the isolated items indicates that all of them have good discriminant capabilities.

The various aspects of the *validity *of the questionnaire that were analyzed produced satisfactory results. The content validity (originally established by a panel of experts) was confirmed by the resulting factor structure. Furthermore, the study of the responses through exploratory and confirmatory factor analysis corroborated the initially proposed theoretical structure and specifically corroborated the presence of six subscales or dimensions: treatment effectiveness, convenience of use, impact on daily activities, medical care, undesired side-effects, and general satisfaction. The relationship detected between the various dimensions suggests that the scores of the different subscales can be combined into a meaningful total score. Whereas we observed a close relationship between the dimensions of effectiveness, impact on daily living, and general satisfaction in the confirmatory analysis, the correlation of the various dimensions with other assessments by patients and the clinician responsible for the treatment suggests that they could actually be considered as different dimensions. It would however be advisable to validate the result in new and larger patient samples.

Multidimensional models similar to the one obtained here have been successfully applied to generic medicine treatments (SATMED-Q) [24] and treatments for asymptomatic diseases such as glaucoma (GLAUSAT) [50], and the dimensions obtained have been shown to be consistent with other models such as the TSQM [18,24]. As a benchmark, we found that the correlation of overall SATMED-Q and TSQM scores was high and positive (r = 0.74), and convergent dimensions of both questionnaires correlated between 0.58 and 0.68.

Comparing the structure obtained for dermatologic treatments with the structure obtained in generic treatments and in glaucoma treatments, several interesting differences are worth mentioning. The number of dimensions and their composition are similar to those obtained for generic treatments, while in asymptomatic treatments as glaucoma an additional dimension covering expectations and beliefs about treatment was needed. Correlations between dimensions (their observed proximity) vary in the three instruments, and we will discuss some of the differences.

In dermatologic treatments *Treatment Effectiveness *is more related to the other dimensions in the questionnaire. In the DERMASAT, *Treatment Effectiveness *highly correlates with *General Satisfaction *(0.92) while the correlation is much lower in generic (0.76) and glaucoma (0.67) treatments. *Treatment Effectiveness *also highly correlates with *Impact on Daily Life *(0.85) as compared to generic (0.75) and glaucoma (0.09) treatments; it has a higher correlation with *Medical Care *(0.53) than generic (0.38) and glaucoma (0.33) treatments; a similar correlation with *Convenience of Use *(0.43) to generic treatments (0.41), but higher than glaucoma (-0.19); and a similar correlation with *Undesired Effects *(-0.18) to generic (-0.12) and glaucoma (-.16).

*Convenience of Use *exhibits a somewhat lower correlation with Impact on Daily Life (0.39) than in generic treatments (0.42) but much lower than in glaucoma (.55); correlates higher with Medical Care (0.42) than in generic (.20) and glaucoma (0.00) treatments; and correlates similarly with General Satisfaction (0.43) to generic treatments (0.45) and higher than in glaucoma ( 0.05).

*Undesired effects *shows a higher correlation with Impact on Daily Life (-0.24) than in generic ( 0.11) and glaucoma (-0.17); lower correlation with General Satisfaction (-0.19) to generic (-0.32) and glaucoma (-0.25); and similar with Medical Care (-0.10) as in generic (-0.12), but lower than in glaucoma (-0.26).

*Medical Care *correlates highly with Impact on Daily Life (0.52) as compared to generic (0.32) and glaucoma (0.30) treatments; and also with General Satisfaction (0.59), as compared to generic (0.35) and glaucoma (0.28).

Summarizing, the structure of patient treatment satisfaction exhibits particular characteristics that need to be considered. Treatment effectiveness is much more related to other aspects of patient satisfaction than in generic and asymptomatic treatments, but it is not confused with undesired effects. Additionally, satisfaction with medical care is also more related to other aspects of satisfaction and its relation with convenience of use and impact on daily life is over-weighted. Undesired effects are clearly distinguished but related to impact on daily life.

From previous results, we expected moderate correlations between satisfaction dimensions and patient reports of compliance, except with undesired effects and medical care. Instead, convenience of use is the only dimension found to have a correlation close to significance with this additional treatment outcome. Hence, usability seems to be the only aspect of treatment satisfaction that could help to forecast treatment compliance. More consistent with theory is the observed correlation between satisfaction with treatment effectiveness and compliance assessed by the clinician. In fact, the correlation between patient and clinician assessment of compliance is only moderate (-0.30), but higher than values obtained for glaucoma treatment (-0.09). These results might reflect the fact that accurate and valid measures of self-reported compliance have yet to be developed, and we will have to wait until such instruments are available before being able to study in detail the relationship between compliance and treatment satisfaction.

On the other hand, effectiveness and tolerability (both assessed by the clinician and the patient) do correlate with satisfaction dimensions and with the overall satisfaction score. Overall satisfaction correlates with clinician and patient effectiveness at a level similar to previous generic questionnaires (0.41 and 0.61, respectively) and higher than in glaucoma (0.27 and 0.19, respectively). Correlations of treatment satisfaction with clinician and patient tolerability assessments are however lower than those found for generic instruments (0.26 and 0.39) and much lower than those found in glaucoma (0.44 and 0.35). Treatment effectiveness seems to be the most influential aspect related to patient satisfaction, while tolerability is a much milder explicative factor.

Differences found between gender groups were not foreseen in advance due to the exploratory nature of the comparison. Nevertheless, they are consistent with other findings from the focus group discussions. Women are more used to regular care of their hands, and don't find it inconvenient to use creams in their daily living, while men (at least in our culture) are not used to it or even show prejudices against using creams.

We expected that more severe disorders would reflect a lower level of satisfaction but results are not conclusive and additional research would be needed. Although the general pattern of means gives some support to this idea (see Figure 2), differences do not reach significance. This could be due to not having enough statistical power owing to small sample size. In fact, 5 out of 9 groups contained fewer than 20 patients, while mean differences are larger than those found between gender groups. But it also could be justified by the fact that there is a very wide range of different treatments in our sample (more than 36 different treatment profiles) and an even wider range of effectiveness experienced by patients.

Regarding concurrent validity, only some of the DermaSat scores correlate with the quality of life and state of health assessments by the patients themselves, suggesting that treatment satisfaction is actually a different type of divergent construct. No relationship is found between the treatment satisfaction assessments and the compliance assessments, something that was not expected in principle, and which requires further research with other groups of patients. Regarding construct validity, we found convergent results when we compared the DermaSat scores with the effectiveness and tolerability assessments (by the clinician and the patient): the greater the perceived effectiveness, the higher the satisfaction scores; the greater the perceived tolerability, the higher the medication-related satisfaction.

As with the SATMED-Q, the DermaSat presents two dimensions of special interest for the patient, one assessing the impact of the medication on activities of daily living and the other assessing patient satisfaction with medical care. These two aspects are highly valued by patients and can serve as an aid to clinicians in making decisions about treatment.

One limitation of this study is that it was cross-sectional and was not capable of examining casual influences of low treatment satisfaction on clinically relevant outcomes. In addition, the responsiveness of this questionnaire to change was not examined in this cross-sectional study. Prospective studies are being planned to address this issue. Another limitation of this study is the fact that no generic instrument measuring satisfaction with treatment was used concurrently. This task was not performed in order to avoid overloading the patients who came to a regular appointment and who were not compensated for their participation in the study. We believe it would be advisable to gather additional data in this regard, a matter that should be easy to tackle now that we have a short questionnaire.

A final limitation of the DermaSat is that the primary validation samples were enrolled in Spain; therefore, the validation of this instrument in international settings is unknown and must be tested.

Our hands are our main 'working tool' and, in some respect, our 'business card' when meeting other people.

The results of our study suggest that treatment satisfaction is very low in this kind of condition, independent on the severity of lesions. All hand lesions, especially if the finger-tips are involved, can severely handicap a patient's daily living when there is no or very low treatment response. This is true for both severe and moderate CHE. This particular combination translates into very low treatment satisfaction with current treatments as found in our study.

On top of this, from a clinician's point of view, correct classification of CHE severity is quite subjective.

It is often the case that mild to moderate hand lesions become chronic and therefore result in severe CHE, even though the lesions per se might not 'look so bad'.

## Conclusions

The DermaSat Questionnaire is a new, specific questionnaire developed to assess the treatment satisfaction in patients with conditions affecting the skin of the hands. The results suggest that the instrument is well accepted by patients and affords good psychometric properties, including the validity and reliability of the subscales that comprise it. The findings support the use of the instrument both in routine clinical practice and in clinical research. The questionnaire will also be useful for assessing new treatment options that might be developed for CHE and comparing them with current treatment options. It would therefore provide an additional decision-making tool, making it possible not to depend only on traditional clinical efficacy data. Moreover, the instrument can contribute to a better understanding of repercussions on the patient of decisions relative to medication, making it an important tool for evaluating the results of the effectiveness of medical care.

## Competing interests

Ralf Halbach and Francisco Regalado are full-time employees of Basilea Pharmaceuticals Iberia SL, the entity providing financial support for the study. The rest of authors declare no competing interest.

## Authors' contributions

The authors of this manuscript state that all of them have contributed substantially in the manuscript preparation, interpretation of results or study design and logistics. MAR, FH, AA, LCS and JC were responsible for the design of the study. MAR and FH carried out the analysis and interpretation of data. FR and RH were responsible of the logistics and conduction of the study. All authors participated in manuscript preparation, and read and approved the final manuscript. FH, AA, LCS, JC, ES and the GEIDAC group were responsible for the recruitment and assessment of patients.

## Supplementary Material

Additional file 1 **Spanish version of the DermaSat questionnaire**. Original Spanish version of the DermaSat questionnaire measuring satisfaction with treatment of hands, used in the validation study. Click here for file

Additional file 2 **English version of the DermaSat questionnaire**. English version of the DermaSat questionnaire measuring satisfaction with treatment of hands. This English version has not been culturally validated and is presented only for reference purposes. Click here for file
