# Supplementary material for: Development and validation of a questionnaire on 'Satisfaction with dermatological treatment of hand eczema' (DermaSat)
Source: Health Qual Life Outcomes. 2010 Nov 5;8:127. doi: 10.1186/1477-7525-8-127 (PMC2992504; doi:10.1186/1477-7525-8-127)
Supplement: Additional file 1 — Spanish version of the DermaSat questionnaire. Original Spanish version of the DermaSat questionnaire measuring satisfaction with treatment of hands, used in the validation study. [file 1477-7525-8-127-S1.DOC]

Additional file 1. Spanish version of the DermaSat questionnaire

**CUESTIONARIO DE SATISFACCIÓN CON EL TRATAMIENTO DE LAS MANOS - DERMASAT**

Deseamos conocer lo contento que está usted con el tratamiento y la medicación que está tomando (cremas hidratantes/emolientes, PUVA, corticoides, otros medicamentos en cremas, antihistaminicos, otro tratamiento oral, etc.) que está siguiendo para el tratamiento de sus manos.

En cada pregunta, **tache** el número que mejor refleja su opinión. No existen respuestas correctas o incorrectas. Si no está seguro de alguna de las respuestas, indique la que le parezca más adecuada.

| - Esta sección se refiere a la **eficacia de la medicación**, es decir, de su capacidad para tratar su enfermedad y aliviar sus síntomas. | | | | |  |
| --- | --- | --- | --- | --- | --- |
|  | Nada | Poco | Bastante | Mucho | |
| 1. La medicación que estoy usando o tomando alivia mis molestias de las manos | ➀ | ➁ | ➂ | ➃ | |
| 1. Me encuentro mejor ahora de lo que me encontraba antes de iniciar el tratamiento | ➀ | ➁ | ➂ | ➃ | |
| 1. Pienso que, actualmente, mi enfermedad de las manos está bien controlada | ➀ | ➁ | ➂ | ➃ | |

| - Esta sección hace referencia a la **comodidad de la medicación** y la facilidad para usarla o tomarla. | | | | | |
| --- | --- | --- | --- | --- | --- |
|  | Nada | Poco | | Bastante | Mucho |
| 1. Me resulta fácil aplicar o tomar el medicamento en su forma actual (textura, olor, tiempo de absorción, sabor, tamaño, etc.) | ➀ | | ➁ | ➂ | ➃ |
| 1. El horario de las tomas o aplicaciones me resulta cómodo | ➀ | | ➁ | ➂ | ➃ |

| - Esta sección hace referencia al **impacto de la medicación** en su vida cotidiana. | | | | | |
| --- | --- | --- | --- | --- | --- |
|  | Nada | Poco | | Bastante | Mucho |
| 1. Gracias a la medicación que estoy tomando puedo realizar mejor mis actividades de tiempo libre | ➀ | | ➁ | ➂ | ➃ |
| 1. Gracias a mi medicación puedo realizar mejor mis tareas cotidianas (aseo personal, tareas de casa, etc.) | ➀ | | ➁ | ➂ | ➃ |
| 1. Gracias a la medicación que estoy usando me encuentro mejor de ánimo | ➀ | | ➁ | ➂ | ➃ |

| - Esta sección se refiere al **seguimiento médico** de su enfermedad. | | | | | |
| --- | --- | --- | --- | --- | --- |
|  | Nada | Poco | | Bastante | Mucho |
| 1. Mi médico me ha informado con detalle acerca de mi enfermedad | ➀ | | ➁ | ➂ | ➃ |
| 1. Mi médico me ha informado acerca de la manera de tratar correctamente mi enfermedad | ➀ | | ➁ | ➂ | ➃ |
| 1. Mi médico me ha informado acerca de los efectos del tratamiento para las manos | ➀ | | ➁ | ➂ | ➃ |

| - Esta sección hace referencia a los **efectos indeseables** producidos por la medicación. | | | | | |
| --- | --- | --- | --- | --- | --- |
|  | Nada | Poco | | Bastante | Mucho |
| 1. Los efectos secundarios de la medicación interfieren en mi actividad física, en mi trabajo o profesión | ➀ | | ➁ | ➂ | ➃ |
| 1. Los efectos indeseables de la medicación interfieren en mis actividades de ocio y tiempo libre | ➀ | | ➁ | ➂ | ➃ |
| 1. Los efectos indeseables de la medicación interfieren en mis tareas cotidianas | ➀ | | ➁ | ➂ | ➃ |

| - Para finalizar, se incluyen unas preguntas acerca de su **opinión general** respecto a la medicación y su estado de salud. | | | | | |
| --- | --- | --- | --- | --- | --- |
|  | Nada | Poco | | Bastante | Mucho |
| 1. Me siento a gusto con mi tratamiento | ➀ | | ➁ | ➂ | ➃ |
| 1. Estoy convencido de que la medicación que estoy tomando es la mejor opción disponible | ➀ | | ➁ | ➂ | ➃ |
| 1. En general, me siento satisfecho con el tratamiento | ➀ | | ➁ | ➂ | ➃ |
