# Supplementary material for: Development and validation of a questionnaire on 'Satisfaction with dermatological treatment of hand eczema' (DermaSat)
Source: Health Qual Life Outcomes. 2010 Nov 5;8:127. doi: 10.1186/1477-7525-8-127 (PMC2992504; doi:10.1186/1477-7525-8-127)
Supplement: Additional file 2 — English version of the DermaSat questionnaire. English version of the DermaSat questionnaire measuring satisfaction with treatment of hands. This English version has not been culturally validated and is presented only for reference purposes. [file 1477-7525-8-127-S2.DOC]

Additonal file 2. English version of the DermaSat questionnaire

**QUESTIONNAIRE ON SATISFACTION WITH TREATMENT OF HANDS - DERMASAT**

We wish to know how satisfied you are with the treatment and medication you are taking (moisturizing / emollient creams, PUVA, corticoids, other medications in cream form, antihistamines, other oral treatment, etc.) that you are following for the care of your hands.

For each question, **cross out** the number that best reflects your opinion. There are no correct or incorrect answers. If you are not sure of any of the answers, indicate the one you think is the most appropriate.

| - This section deals with the **effectiveness of the medication**, that is, its ability to treat your condition and alleviate its symptoms. | | | | |
| --- | --- | --- | --- | --- |
|  | Not at all | A Little | Enough | A lot |
| 1. The medication I am using or taking relieves the discomfort my hands give me | ➀ | ➁ | ➂ | ➃ |
| 1. I feel better now than I did before starting the treatment | ➀ | ➁ | ➂ | ➃ |
| 1. I think that my skin disease is now well under control | ➀ | ➁ | ➂ | ➃ |

| - This section makes reference to **how convenient the medication is** and how easy it is to use or take it. | | | | |
| --- | --- | --- | --- | --- |
|  | Not at all | A Little | Enough | A lot |
| 1. I find it easy to apply or take the medication in its present form (texture, odour, absorption time, flavour, size, etc.) | ➀ | ➁ | ➂ | ➃ |
| 1. The schedule for taking or applying the medication is convenient for me | ➀ | ➁ | ➂ | ➃ |

| - This section deals with the **impact of the medication** on your daily life. | | | | |
| --- | --- | --- | --- | --- |
|  | Not at all | A Little | Enough | A lot |
| 1. Thanks to the medication I am taking, I can do my free time activities better | ➀ | ➁ | ➂ | ➃ |
| 1. Thanks to my medication, I can perform my daily tasks better (personal hygiene, housework, etc.) | ➀ | ➁ | ➂ | ➃ |
| 1. Thanks to the medication I am using I am in a better mood | ➀ | ➁ | ➂ | ➃ |

| - This section makes reference to the **medical follow-up** on your condition. | | | | |
| --- | --- | --- | --- | --- |
|  | Not at all | A Little | Enough | A lot |
| 1. My doctor gave me detailed information about my condition | ➀ | ➁ | ➂ | ➃ |
| 1. My doctor informed me about the correct way to treat my condition | ➀ | ➁ | ➂ | ➃ |
| 1. My doctor informed me of the effects of the treatment for my hands | ➀ | ➁ | ➂ | ➃ |

| - This section is about the **undesirable effects** produced by the medication. | | | | |
| --- | --- | --- | --- | --- |
|  | Not at all | A Little | Enough | A lot |
| 1. The side effects of the medication interfere with my physical activity, my work or occupation | ➀ | ➁ | ➂ | ➃ |
| 1. The undesirable effects of the medication interfere with my leisure and free time activities | ➀ | ➁ | ➂ | ➃ |
| 1. The undesirable effects of the medication interfere with my daily tasks | ➀ | ➁ | ➂ | ➃ |

| - Finally, some questions are included about your **general opinion** of the medication and your state of health. | | | | |
| --- | --- | --- | --- | --- |
|  | Not at all | A Little | Enough | A lot |
| 1. I feel satisfied with the treatment | ➀ | ➁ | ➂ | ➃ |
| 1. I am convinced that the medication I am taking is the best option available | ➀ | ➁ | ➂ | ➃ |
| 1. In general, I feel satisfied with the treatment | ➀ | ➁ | ➂ | ➃ |

Note: This English version has not been culturally validated and is presented only for reference purposes.
